# Diethyl 2-[(2-benzyl-1-phenyl­sulfonyl-1*H*-indol-3-yl)methyl­ene]malonate

**DOI:** 10.1107/S160053680804004X

**Published:** 2008-12-03

**Authors:** T. Kavitha, M. Thenmozhi, V. Dhayalan, A. K. Mohanakrishnan, M. N. Ponnuswamy

**Affiliations:** a Centre of Advanced Study in Crystallography and Biophysics, University of Madras, Guindy Campus, Chennai 600025, India; b Department of Organic Chemistry, University of Madras, Guindy Campus, Chennai 600025, India

## Abstract

In the title compound, C_29_H_27_NO_6_S, the sulfonyl-bound phenyl ring is almost perpendicular to the indole ring system [dihedral angle = 87.96 (6)°], while the benzyl­phenyl ring is oriented at an angle of 76.8 (7)°. An intra­molecular C—H⋯O hydrogen bond is observed. In the crystal structure, mol­ecules are linked into a zigzag *C*(10) chain along the *b* axis by inter­molecular C—H⋯O hydrogen bonds.

## Related literature

For general background to indole derivatives and their biological activity, see: Andreani *et al.* (2001 ▶); Bassindale (1984 ▶); Quetin-Leclercq *et al.* (1995 ▶); Singh *et al.* (2000 ▶); Tsotinis *et al.* (1997 ▶); Wang & Ng (2002 ▶).
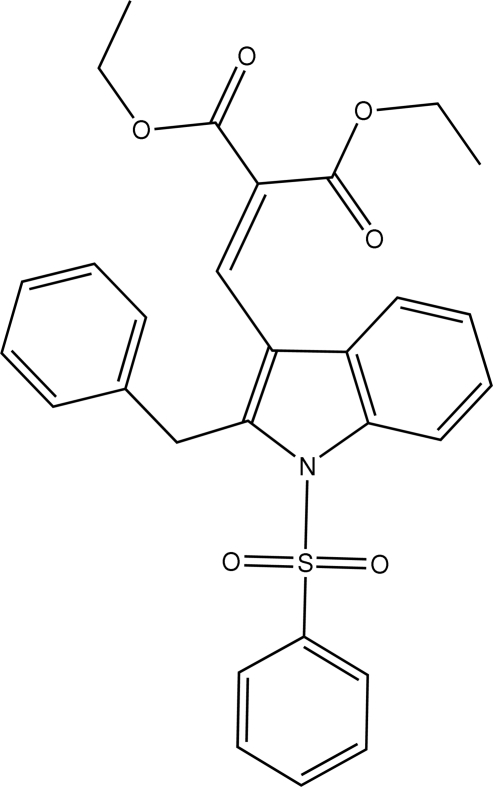


## Experimental

### 

#### Crystal data

C_29_H_27_NO_6_S
*M* *_r_* = 517.58
Monoclinic, 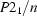

*a* = 10.8280 (9) Å
*b* = 13.7762 (11) Å
*c* = 17.6832 (16) Å
β = 91.341 (4)°
*V* = 2637.1 (4) Å^3^
*Z* = 4
Mo *K*α radiation
μ = 0.17 mm^−1^
*T* = 293 (2) K
0.30 × 0.25 × 0.16 mm

#### Data collection

Bruker Kappa APEXII area-detector diffractometer
Absorption correction: multi-scan (*SADABS*; Sheldrick, 2001 ▶) *T*_min_ = 0.952, *T*_max_ = 0.968
34168 measured reflections
7583 independent reflections
4683 reflections with *I* > 2σ(*I*)
*R*_int_ = 0.030

#### Refinement

*R*[*F* ^2^ > 2σ(*F* ^2^)] = 0.048
*wR*(*F* ^2^) = 0.142
*S* = 1.02
7583 reflections
334 parameters
1 restraint
H-atom parameters constrained
Δρ_max_ = 0.25 e Å^−3^
Δρ_min_ = −0.27 e Å^−3^

### 

Data collection: *APEX2* (Bruker, 2004 ▶); cell refinement: *APEX2*; data reduction: *SAINT* (Bruker, 2004 ▶); program(s) used to solve structure: *SHELXS97* (Sheldrick, 2008 ▶); program(s) used to refine structure: *SHELXL97* (Sheldrick, 2008 ▶); molecular graphics: *ORTEP-3* (Farrugia, 1997 ▶); software used to prepare material for publication: *SHELXL97* and *PLATON* (Spek, 2003 ▶).

## Supplementary Material

Crystal structure: contains datablocks I, global. DOI: 10.1107/S160053680804004X/ci2729sup1.cif

Structure factors: contains datablocks I. DOI: 10.1107/S160053680804004X/ci2729Isup2.hkl

Additional supplementary materials:  crystallographic information; 3D view; checkCIF report

## Figures and Tables

**Table 1:** Hydrogen-bond geometry (Å, °)

*D*—H⋯*A*	*D*—H	H⋯*A*	*D*⋯*A*	*D*—H⋯*A*
C6—H6⋯O1	0.93	2.33	2.919 (2)	121
C7—H7⋯O5^i^	0.93	2.59	3.317 (2)	135

Symmetry code: (i) 
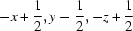
.

**Fig. 1.**
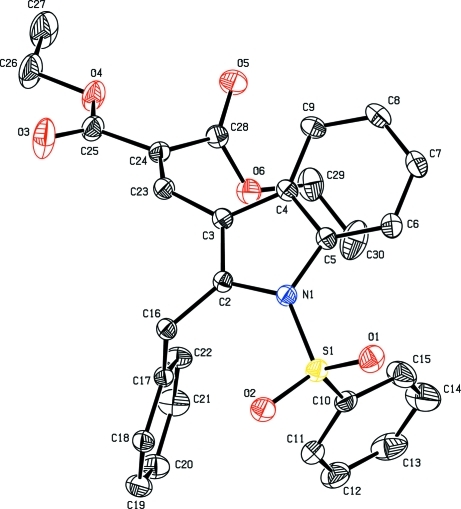
The molecular structure of the title compound. Displacement ellipsoids are drawn at the 20% probability level. H atoms have been omitted for clarity.

**Fig. 2.**
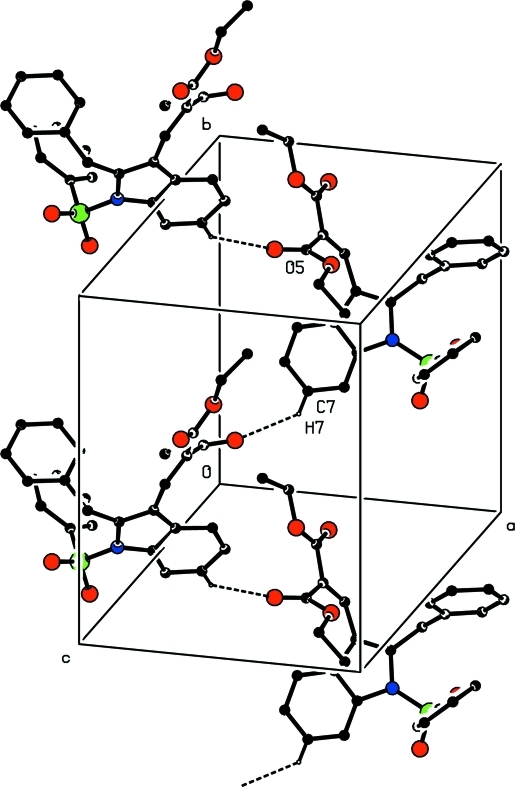
Crystal packing of the title compound. H atoms not involved in hydrogen bonding have been omitted.

## supplementary crystallographic information

### Comment

Indole derivatives have been found to exhibit antibacterial, antifungal (Wang & Ng, 2002; Singh *et al.*, 2000; Tsotinis *et al.*, 1997; Quetin-Leclercq *et al.*, 1995) and antitumour activities (Andreani *et al.*, 2001).

The longer C—N distances [N1—C2 = 1.417 (2) Å and N1—C5 = 1.421 (2) Å] are indicative of the electronic withdrawing character of the phenylsulfonyl group. Atom S1 has a distorted tetrahedral configuration. The widening of the O1—S1—O2 angle to 120.09 (8)°, and the resultant narrowing of the N1—S1—C10 to 104.99 (8)°, from the ideal tetrahedral values, are attributed to the Thorpe- Ingold effect (Bassindale, 1984). The indole ring system is planar. The sum of bond angles around N1 (358.0°) indicates that N1 is in *sp*^2^ hybridization. The sulfonyl bound phenyl ring is almost perpendicular to the indole ring system, with a dihedral angle of 87.96 (6)°. The benzylphenyl ring is oriented at an angle of 76.69 (5)° with respect to the indole ring system. The ester groups attached to the indole ring system assume extended conformations [C3—C23—C24—C25 = 178.7 (2)°, C23—C24—C25—O4 = -176.8 (2)°, C24—C25—O4—C26 =179.4 (2)°, C25—O4—C26—C27 = 172.6 (2)°]. An intramolecular C6—H6···O1 hydrogen bond is observed.

In the crystal structure, the molecules are linked into a zigzag C(10) chain along the *b* axis by intermolecular C—H···O hydrogen bonds (Table 1 and Fig.2).

### Experimental

To a solution of diethyl-2-((2-(bromomethyl)-1-(phenylsulfonyl)-1*H*-indol-3-yl)methylene)malonate (0.5 g, 0.96 mmol) in dry benzene (15 ml), ZnBr_2_ (0.43 g, 1.90 mmol) was added. The reaction mixture was then refluxed for 5 h under N_2_ atmosphere. It was then poured over ice-water (50 ml) containing 1 ml of concentrated HCl, extracted with chloroform (2 × 10 ml) and dried (Na_2_SO_4_). Removal of solvent followed by flash column chromatography (silica gel, 230–420 mesh, *n*-hexane/ethyl acetate 99:1) led to the isolation of diethyl-2-((2-benzyl-1-(phenylsulfonyl)-1*H*-indol-3-yl)methylene)malonate as a colourless solid. Single crystals were obtained by recrystallization from CDCl_3_.

### Refinement

H atoms were positioned geometrically (C—H = 0.93–0.98 Å) and allowed to ride on their parent atoms, with *U*_iso_(H) = 1.5*U*_eq_(C_methyl_) and 1.2*U*_eq_(C). The components of the anisotropic displacement parameters of C26 and C27 in the direction of the bond between them were restrained to be equal within an effective standard deviation of 0.001.

### Crystal data

**Table:**

C_29_H_27_NO_6_S	*F*(000) = 1088
*M**_r_* = 517.58	*D*_x_ = 1.304 Mg m^−^^3^
Monoclinic, *P*2_1_/*n*	Mo *K*α radiation, λ = 0.71073 Å
Hall symbol: -P 2yn	Cell parameters from 7583 reflections
*a* = 10.8280 (9) Å	θ = 2.2–29.9°
*b* = 13.7762 (11) Å	µ = 0.17 mm^−^^1^
*c* = 17.6832 (16) Å	*T* = 293 K
β = 91.341 (4)°	Block, colourless
*V* = 2637.1 (4) Å^3^	0.30 × 0.25 × 0.16 mm
*Z* = 4	

### Data collection

**Table:**

Bruker Kappa APEXII area-detector diffractometer	7583 independent reflections
Radiation source: fine-focus sealed tube	4683 reflections with *I* > 2σ(*I*)
graphite	*R*_int_ = 0.030
ω and φ scans	θ_max_ = 29.9°, θ_min_ = 2.2°
Absorption correction: multi-scan (*SADABS*; Sheldrick, 2001)	*h* = −15→12
*T*_min_ = 0.952, *T*_max_ = 0.968	*k* = −14→19
34168 measured reflections	*l* = −22→24

### Refinement

**Table:**

Refinement on *F*^2^	Primary atom site location: structure-invariant direct methods
Least-squares matrix: full	Secondary atom site location: difference Fourier map
*R*[*F*^2^ > 2σ(*F*^2^)] = 0.048	Hydrogen site location: inferred from neighbouring sites
*wR*(*F*^2^) = 0.142	H-atom parameters constrained
*S* = 1.02	*w* = 1/[σ^2^(*F*_o_^2^) + (0.0628*P*)^2^ + 0.5537*P*] where *P* = (*F*_o_^2^ + 2*F*_c_^2^)/3
7583 reflections	(Δ/σ)_max_ = 0.021
334 parameters	Δρ_max_ = 0.25 e Å^−^^3^
1 restraint	Δρ_min_ = −0.27 e Å^−^^3^

### Special details

**Table:**

Geometry. All e.s.d.'s (except the e.s.d. in the dihedral angle between two l.s. planes) are estimated using the full covariance matrix. The cell e.s.d.'s are taken into account individually in the estimation of e.s.d.'s in distances, angles and torsion angles; correlations between e.s.d.'s in cell parameters are only used when they are defined by crystal symmetry. An approximate (isotropic) treatment of cell e.s.d.'s is used for estimating e.s.d.'s involving l.s. planes.
Refinement. Refinement of *F*^2^ against ALL reflections. The weighted *R*-factor *wR* and goodness of fit *S* are based on *F*^2^, conventional *R*-factors *R* are based on *F*, with *F* set to zero for negative *F*^2^. The threshold expression of *F*^2^ > σ(*F*^2^) is used only for calculating *R*-factors(gt) *etc*. and is not relevant to the choice of reflections for refinement. *R*-factors based on *F*^2^ are statistically about twice as large as those based on *F*, and *R*- factors based on ALL data will be even larger.

### Fractional atomic coordinates and isotropic or equivalent isotropic displacement parameters (Å^2^)

**Table:**

	*x*	*y*	*z*	*U*_iso_*/*U*_eq_	
C2	0.62844 (14)	0.59182 (11)	0.04158 (8)	0.0397 (3)	
C3	0.51591 (14)	0.62397 (12)	0.06343 (8)	0.0413 (3)	
C4	0.46420 (15)	0.55336 (12)	0.11419 (8)	0.0429 (3)	
C5	0.54970 (15)	0.47797 (12)	0.12226 (8)	0.0433 (4)	
C6	0.52568 (17)	0.39664 (14)	0.16628 (10)	0.0546 (4)	
H6	0.5824	0.3462	0.1707	0.066*	
C7	0.41513 (19)	0.39360 (15)	0.20291 (10)	0.0612 (5)	
H7	0.3981	0.3409	0.2338	0.073*	
C8	0.32872 (18)	0.46698 (15)	0.19496 (10)	0.0597 (5)	
H8	0.2546	0.4624	0.2202	0.072*	
C9	0.35069 (16)	0.54695 (13)	0.15017 (10)	0.0524 (4)	
H9	0.2915	0.5954	0.1440	0.063*	
C10	0.86326 (16)	0.49835 (14)	0.16366 (10)	0.0524 (4)	
C11	0.96281 (18)	0.55897 (16)	0.15389 (12)	0.0648 (5)	
H11	0.9927	0.5708	0.1059	0.078*	
C12	1.0176 (2)	0.60210 (19)	0.21722 (15)	0.0835 (7)	
H12	1.0852	0.6430	0.2118	0.100*	
C13	0.9725 (2)	0.5846 (2)	0.28747 (16)	0.0986 (9)	
H13	1.0073	0.6158	0.3294	0.118*	
C14	0.8760 (3)	0.5212 (3)	0.29651 (13)	0.1091 (10)	
H14	0.8478	0.5080	0.3447	0.131*	
C15	0.8210 (2)	0.4773 (2)	0.23443 (12)	0.0856 (7)	
H15	0.7560	0.4340	0.2403	0.103*	
C16	0.71395 (15)	0.64140 (12)	−0.01088 (8)	0.0446 (4)	
H16A	0.6652	0.6821	−0.0449	0.053*	
H16B	0.7534	0.5923	−0.0414	0.053*	
C17	0.81366 (14)	0.70333 (12)	0.02598 (9)	0.0426 (3)	
C18	0.93068 (15)	0.70523 (13)	−0.00413 (10)	0.0510 (4)	
H18	0.9470	0.6673	−0.0462	0.061*	
C19	1.02346 (18)	0.76232 (15)	0.02708 (12)	0.0647 (5)	
H19	1.1014	0.7624	0.0061	0.078*	
C20	1.00051 (19)	0.81899 (17)	0.08912 (13)	0.0724 (6)	
H20	1.0630	0.8570	0.1107	0.087*	
C21	0.8847 (2)	0.81906 (17)	0.11901 (12)	0.0741 (6)	
H21	0.8686	0.8581	0.1605	0.089*	
C22	0.79141 (16)	0.76163 (14)	0.08807 (10)	0.0567 (5)	
H22	0.7135	0.7622	0.1091	0.068*	
C23	0.46256 (15)	0.71538 (13)	0.03676 (9)	0.0477 (4)	
H23	0.4694	0.7277	−0.0147	0.057*	
C24	0.40536 (15)	0.78361 (12)	0.07624 (9)	0.0476 (4)	
C25	0.36165 (19)	0.87182 (15)	0.03489 (12)	0.0619 (5)	
C26	0.2677 (3)	1.02592 (18)	0.04540 (15)	0.1004 (9)	
H26A	0.3365	1.0636	0.0270	0.120*	
H26B	0.2122	1.0115	0.0030	0.120*	
C27	0.2032 (3)	1.0801 (2)	0.10279 (18)	0.1203 (12)	
H27A	0.1723	1.1397	0.0814	0.180*	
H27B	0.2591	1.0943	0.1442	0.180*	
H27C	0.1355	1.0421	0.1206	0.180*	
C28	0.38732 (16)	0.77720 (12)	0.15970 (10)	0.0502 (4)	
C29	0.4911 (2)	0.74032 (19)	0.27630 (11)	0.0809 (7)	
H29A	0.4125	0.7117	0.2892	0.097*	
H29B	0.5012	0.8004	0.3043	0.097*	
C30	0.5929 (3)	0.6728 (2)	0.29586 (14)	0.1064 (9)	
H30A	0.5924	0.6590	0.3491	0.160*	
H30B	0.6703	0.7020	0.2834	0.160*	
H30C	0.5821	0.6136	0.2679	0.160*	
N1	0.65221 (12)	0.50118 (10)	0.07694 (7)	0.0434 (3)	
O1	0.76255 (13)	0.34702 (9)	0.10092 (8)	0.0690 (4)	
O2	0.85555 (12)	0.46945 (10)	0.01832 (7)	0.0633 (4)	
O3	0.3719 (2)	0.88321 (13)	−0.03157 (9)	0.1094 (7)	
O4	0.31220 (15)	0.93642 (10)	0.08018 (8)	0.0764 (4)	
O5	0.29041 (13)	0.78452 (13)	0.18992 (8)	0.0796 (4)	
O6	0.49406 (12)	0.75941 (10)	0.19517 (6)	0.0585 (3)	
S1	0.78964 (4)	0.44550 (3)	0.08437 (2)	0.04989 (13)	

### Atomic displacement parameters (Å^2^)

**Table:**

	*U*^11^	*U*^22^	*U*^33^	*U*^12^	*U*^13^	*U*^23^
C2	0.0459 (8)	0.0424 (8)	0.0309 (7)	0.0023 (7)	0.0020 (6)	−0.0038 (6)
C3	0.0452 (8)	0.0447 (8)	0.0340 (7)	0.0016 (7)	0.0032 (6)	−0.0048 (6)
C4	0.0464 (8)	0.0455 (9)	0.0370 (8)	−0.0037 (7)	0.0048 (6)	−0.0059 (7)
C5	0.0489 (9)	0.0456 (9)	0.0355 (7)	−0.0019 (7)	0.0027 (6)	−0.0039 (6)
C6	0.0612 (11)	0.0529 (10)	0.0498 (10)	−0.0033 (8)	0.0006 (8)	0.0058 (8)
C7	0.0703 (12)	0.0621 (12)	0.0514 (10)	−0.0159 (10)	0.0063 (9)	0.0086 (9)
C8	0.0590 (11)	0.0694 (13)	0.0512 (10)	−0.0182 (10)	0.0143 (8)	−0.0059 (9)
C9	0.0486 (9)	0.0565 (10)	0.0524 (10)	−0.0037 (8)	0.0107 (8)	−0.0101 (8)
C10	0.0457 (9)	0.0609 (11)	0.0506 (9)	0.0128 (8)	0.0012 (7)	0.0029 (8)
C11	0.0536 (11)	0.0751 (13)	0.0653 (12)	0.0044 (10)	−0.0044 (9)	0.0152 (10)
C12	0.0632 (13)	0.0920 (17)	0.0942 (18)	−0.0040 (12)	−0.0210 (13)	0.0058 (14)
C13	0.0718 (16)	0.141 (2)	0.0823 (18)	0.0089 (16)	−0.0203 (13)	−0.0306 (17)
C14	0.0797 (17)	0.195 (3)	0.0526 (13)	−0.012 (2)	0.0023 (12)	−0.0129 (17)
C15	0.0687 (13)	0.137 (2)	0.0510 (12)	−0.0199 (14)	0.0061 (10)	−0.0014 (13)
C16	0.0485 (9)	0.0516 (9)	0.0338 (7)	0.0050 (7)	0.0063 (6)	−0.0001 (7)
C17	0.0439 (8)	0.0426 (8)	0.0415 (8)	0.0067 (7)	0.0062 (6)	0.0043 (7)
C18	0.0505 (9)	0.0530 (10)	0.0499 (9)	0.0071 (8)	0.0113 (7)	0.0025 (8)
C19	0.0500 (10)	0.0672 (13)	0.0775 (13)	−0.0016 (9)	0.0109 (9)	0.0031 (11)
C20	0.0570 (11)	0.0733 (14)	0.0868 (15)	−0.0107 (10)	−0.0022 (10)	−0.0109 (12)
C21	0.0729 (13)	0.0775 (15)	0.0720 (13)	−0.0009 (11)	0.0059 (11)	−0.0294 (11)
C22	0.0480 (9)	0.0650 (12)	0.0575 (10)	0.0042 (8)	0.0101 (8)	−0.0138 (9)
C23	0.0488 (9)	0.0541 (10)	0.0405 (8)	0.0058 (8)	0.0053 (7)	0.0017 (7)
C24	0.0456 (8)	0.0496 (9)	0.0477 (9)	0.0084 (7)	0.0050 (7)	0.0033 (7)
C25	0.0677 (12)	0.0595 (11)	0.0588 (11)	0.0184 (10)	0.0064 (9)	0.0085 (9)
C26	0.132 (2)	0.0731 (16)	0.0966 (17)	0.0495 (15)	0.0213 (15)	0.0311 (13)
C27	0.153 (3)	0.0816 (18)	0.128 (2)	0.0647 (19)	0.038 (2)	0.0285 (16)
C28	0.0561 (10)	0.0433 (9)	0.0516 (9)	0.0084 (8)	0.0111 (8)	−0.0023 (7)
C29	0.1101 (18)	0.0912 (16)	0.0414 (10)	−0.0064 (14)	−0.0021 (11)	−0.0033 (10)
C30	0.152 (3)	0.098 (2)	0.0674 (15)	0.0107 (19)	−0.0347 (16)	0.0001 (14)
N1	0.0451 (7)	0.0462 (7)	0.0392 (7)	0.0041 (6)	0.0041 (5)	0.0004 (6)
O1	0.0787 (9)	0.0475 (7)	0.0809 (9)	0.0133 (7)	0.0053 (7)	0.0002 (7)
O2	0.0642 (8)	0.0748 (9)	0.0517 (7)	0.0226 (7)	0.0178 (6)	−0.0014 (6)
O3	0.1778 (19)	0.0924 (12)	0.0589 (10)	0.0551 (13)	0.0223 (11)	0.0235 (8)
O4	0.0987 (11)	0.0608 (9)	0.0703 (9)	0.0373 (8)	0.0117 (8)	0.0129 (7)
O5	0.0693 (9)	0.1000 (12)	0.0707 (9)	0.0266 (8)	0.0279 (7)	0.0055 (8)
O6	0.0627 (8)	0.0694 (8)	0.0434 (6)	−0.0004 (6)	0.0007 (6)	−0.0052 (6)
S1	0.0533 (3)	0.0490 (3)	0.0476 (2)	0.01314 (19)	0.00624 (18)	−0.00205 (18)

### Geometric parameters (Å, °)

**Table:**

C2—C3	1.361 (2)	C18—H18	0.93
C2—N1	1.417 (2)	C19—C20	1.374 (3)
C2—C16	1.492 (2)	C19—H19	0.93
C3—C4	1.445 (2)	C20—C21	1.372 (3)
C3—C23	1.459 (2)	C20—H20	0.93
C4—C5	1.396 (2)	C21—C22	1.386 (3)
C4—C9	1.400 (2)	C21—H21	0.93
C5—C6	1.392 (2)	C22—H22	0.93
C5—N1	1.421 (2)	C23—C24	1.332 (2)
C6—C7	1.375 (3)	C23—H23	0.93
C6—H6	0.93	C24—C25	1.490 (2)
C7—C8	1.382 (3)	C24—C28	1.496 (2)
C7—H7	0.93	C25—O3	1.193 (2)
C8—C9	1.381 (3)	C25—O4	1.319 (2)
C8—H8	0.93	C26—C27	1.452 (4)
C9—H9	0.93	C26—O4	1.455 (2)
C10—C15	1.373 (3)	C26—H26A	0.97
C10—C11	1.378 (3)	C26—H26B	0.97
C10—S1	1.7548 (18)	C27—H27A	0.96
C11—C12	1.389 (3)	C27—H27B	0.96
C11—H11	0.93	C27—H27C	0.96
C12—C13	1.367 (4)	C28—O5	1.193 (2)
C12—H12	0.93	C28—O6	1.325 (2)
C13—C14	1.373 (4)	C29—O6	1.460 (2)
C13—H13	0.93	C29—C30	1.477 (4)
C14—C15	1.377 (3)	C29—H29A	0.97
C14—H14	0.93	C29—H29B	0.97
C15—H15	0.93	C30—H30A	0.96
C16—C17	1.512 (2)	C30—H30B	0.96
C16—H16A	0.97	C30—H30C	0.96
C16—H16B	0.97	N1—S1	1.6766 (13)
C17—C18	1.386 (2)	O1—S1	1.4199 (14)
C17—C22	1.386 (2)	O2—S1	1.4216 (13)
C18—C19	1.381 (3)		
			
C3—C2—N1	108.43 (13)	C21—C20—C19	119.47 (19)
C3—C2—C16	126.91 (14)	C21—C20—H20	120.3
N1—C2—C16	124.67 (13)	C19—C20—H20	120.3
C2—C3—C4	108.64 (14)	C20—C21—C22	120.78 (19)
C2—C3—C23	122.64 (15)	C20—C21—H21	119.6
C4—C3—C23	128.71 (14)	C22—C21—H21	119.6
C5—C4—C9	119.58 (15)	C21—C22—C17	120.34 (16)
C5—C4—C3	107.37 (14)	C21—C22—H22	119.8
C9—C4—C3	132.97 (16)	C17—C22—H22	119.8
C6—C5—C4	121.58 (15)	C24—C23—C3	128.67 (15)
C6—C5—N1	130.93 (16)	C24—C23—H23	115.7
C4—C5—N1	107.45 (13)	C3—C23—H23	115.7
C7—C6—C5	117.64 (18)	C23—C24—C25	117.68 (16)
C7—C6—H6	121.2	C23—C24—C28	123.27 (15)
C5—C6—H6	121.2	C25—C24—C28	119.02 (15)
C6—C7—C8	121.64 (18)	O3—C25—O4	123.89 (18)
C6—C7—H7	119.2	O3—C25—C24	123.70 (18)
C8—C7—H7	119.2	O4—C25—C24	112.41 (16)
C9—C8—C7	121.09 (17)	C27—C26—O4	107.5 (2)
C9—C8—H8	119.5	C27—C26—H26A	110.2
C7—C8—H8	119.5	O4—C26—H26A	110.2
C8—C9—C4	118.42 (17)	C27—C26—H26B	110.2
C8—C9—H9	120.8	O4—C26—H26B	110.2
C4—C9—H9	120.8	H26A—C26—H26B	108.5
C15—C10—C11	121.37 (19)	C26—C27—H27A	109.5
C15—C10—S1	119.07 (16)	C26—C27—H27B	109.5
C11—C10—S1	119.56 (15)	H27A—C27—H27B	109.5
C10—C11—C12	118.6 (2)	C26—C27—H27C	109.5
C10—C11—H11	120.7	H27A—C27—H27C	109.5
C12—C11—H11	120.7	H27B—C27—H27C	109.5
C13—C12—C11	120.2 (2)	O5—C28—O6	124.78 (17)
C13—C12—H12	119.9	O5—C28—C24	124.99 (17)
C11—C12—H12	119.9	O6—C28—C24	110.20 (14)
C12—C13—C14	120.5 (2)	O6—C29—C30	108.07 (19)
C12—C13—H13	119.8	O6—C29—H29A	110.1
C14—C13—H13	119.8	C30—C29—H29A	110.1
C13—C14—C15	120.2 (2)	O6—C29—H29B	110.1
C13—C14—H14	119.9	C30—C29—H29B	110.1
C15—C14—H14	119.9	H29A—C29—H29B	108.4
C10—C15—C14	119.1 (2)	C29—C30—H30A	109.5
C10—C15—H15	120.4	C29—C30—H30B	109.5
C14—C15—H15	120.4	H30A—C30—H30B	109.5
C2—C16—C17	115.95 (12)	C29—C30—H30C	109.5
C2—C16—H16A	108.3	H30A—C30—H30C	109.5
C17—C16—H16A	108.3	H30B—C30—H30C	109.5
C2—C16—H16B	108.3	C2—N1—C5	108.11 (12)
C17—C16—H16B	108.3	C2—N1—S1	126.11 (11)
H16A—C16—H16B	107.4	C5—N1—S1	123.82 (11)
C18—C17—C22	118.08 (16)	C25—O4—C26	116.75 (17)
C18—C17—C16	119.68 (14)	C28—O6—C29	117.36 (16)
C22—C17—C16	122.21 (14)	O1—S1—O2	120.08 (9)
C19—C18—C17	121.36 (17)	O1—S1—N1	105.43 (8)
C19—C18—H18	119.3	O2—S1—N1	106.92 (7)
C17—C18—H18	119.3	O1—S1—C10	108.90 (9)
C20—C19—C18	119.95 (18)	O2—S1—C10	109.44 (9)
C20—C19—H19	120.0	N1—S1—C10	104.98 (7)
C18—C19—H19	120.0		
			
N1—C2—C3—C4	0.01 (17)	C3—C2—N1—S1	−164.69 (11)
C16—C2—C3—C4	179.63 (14)	C16—C2—N1—S1	15.7 (2)
N1—C2—C3—C23	−179.53 (13)	C6—C5—N1—C2	177.89 (16)
C16—C2—C3—C23	0.1 (2)	C4—C5—N1—C2	0.36 (16)
C2—C3—C4—C5	0.22 (17)	C6—C5—N1—S1	−17.2 (2)
C23—C3—C4—C5	179.72 (15)	C4—C5—N1—S1	165.27 (11)
C2—C3—C4—C9	−176.51 (17)	O4—C25—O3—O3	0.0 (3)
C23—C3—C4—C9	3.0 (3)	C24—C25—O3—O3	0.0 (3)
C9—C4—C5—C6	−0.9 (2)	O3—C25—O4—C26	0.4 (4)
C3—C4—C5—C6	−178.16 (14)	O3—C25—O4—C26	0.4 (4)
C9—C4—C5—N1	176.89 (14)	C24—C25—O4—C26	179.4 (2)
C3—C4—C5—N1	−0.35 (17)	C27—C26—O4—C25	172.6 (2)
C4—C5—C6—C7	−1.1 (2)	O5—C28—O6—C29	4.8 (3)
N1—C5—C6—C7	−178.33 (16)	C24—C28—O6—C29	−173.44 (16)
C5—C6—C7—C8	1.8 (3)	C30—C29—O6—C28	148.87 (19)
C6—C7—C8—C9	−0.5 (3)	O1—O1—S1—O2	0.00 (10)
C7—C8—C9—C4	−1.5 (3)	O1—O1—S1—O2	0.00 (10)
C5—C4—C9—C8	2.2 (2)	O1—O1—S1—O2	0.00 (10)
C3—C4—C9—C8	178.61 (16)	O1—O1—S1—N1	0.00 (9)
C15—C10—C11—C12	−2.5 (3)	O1—O1—S1—C10	0.00 (10)
S1—C10—C11—C12	178.27 (16)	O2—O2—S1—O1	0.00 (6)
C10—C11—C12—C13	−0.3 (3)	O2—O2—S1—O1	0.00 (6)
C11—C12—C13—C14	2.7 (4)	O2—O2—S1—O1	0.00 (6)
C12—C13—C14—C15	−2.3 (5)	O2—O2—S1—O1	0.00 (6)
C11—C10—C15—C14	2.9 (4)	O2—O2—S1—O2	0.00 (4)
S1—C10—C15—C14	−177.9 (2)	O2—O2—S1—O2	0.00 (4)
C13—C14—C15—C10	−0.5 (5)	O2—O2—S1—N1	0.00 (2)
C3—C2—C16—C17	95.29 (18)	O2—O2—S1—N1	0.00 (2)
N1—C2—C16—C17	−85.15 (19)	O2—O2—S1—C10	0.00 (5)
C2—C16—C17—C22	−40.0 (2)	O2—O2—S1—C10	0.00 (5)
C2—C16—C17—C18	142.20 (15)	C2—N1—S1—O1	−161.77 (13)
C22—C17—C18—C19	0.9 (3)	C5—N1—S1—O1	36.07 (15)
C16—C17—C18—C19	178.73 (16)	C2—N1—S1—O1	−161.77 (13)
C17—C18—C19—C20	−0.2 (3)	C5—N1—S1—O1	36.07 (15)
C18—C19—C20—C21	−0.7 (3)	C2—N1—S1—O2	−32.90 (15)
C19—C20—C21—C22	1.0 (4)	C5—N1—S1—O2	164.94 (13)
C20—C21—C22—C17	−0.3 (3)	C2—N1—S1—O2	−32.90 (15)
C18—C17—C22—C21	−0.6 (3)	C5—N1—S1—O2	164.94 (13)
C16—C17—C22—C21	−178.43 (18)	C2—N1—S1—O2	−32.90 (15)
C2—C3—C23—C24	−135.86 (19)	C5—N1—S1—O2	164.94 (13)
C4—C3—C23—C24	44.7 (3)	C2—N1—S1—C10	83.29 (14)
C3—C23—C24—C25	178.68 (17)	C5—N1—S1—C10	−78.86 (14)
C3—C23—C24—C28	0.9 (3)	C15—C10—S1—O1	−41.3 (2)
C23—C24—C25—O3	2.2 (3)	C11—C10—S1—O1	137.98 (16)
C28—C24—C25—O3	−179.9 (2)	C15—C10—S1—O1	−41.3 (2)
C23—C24—C25—O3	2.2 (3)	C11—C10—S1—O1	137.98 (16)
C28—C24—C25—O3	−179.9 (2)	C15—C10—S1—O2	−174.34 (17)
C23—C24—C25—O4	−176.75 (17)	C11—C10—S1—O2	4.93 (18)
C28—C24—C25—O4	1.1 (3)	C15—C10—S1—O2	−174.34 (17)
C23—C24—C28—O5	−126.2 (2)	C11—C10—S1—O2	4.93 (18)
C25—C24—C28—O5	56.1 (3)	C15—C10—S1—O2	−174.34 (17)
C23—C24—C28—O6	52.0 (2)	C11—C10—S1—O2	4.93 (18)
C25—C24—C28—O6	−125.73 (17)	C15—C10—S1—N1	71.20 (19)
C3—C2—N1—C5	−0.23 (16)	C11—C10—S1—N1	−109.53 (15)
C16—C2—N1—C5	−179.86 (13)		

### Hydrogen-bond geometry (Å, °)

**Table:**

*D*—H···*A*	*D*—H	H···*A*	*D*···*A*	*D*—H···*A*
C6—H6···O1	0.93	2.33	2.919 (2)	121
C7—H7···O5^i^	0.93	2.59	3.317 (2)	135

Symmetry codes:  (i) −*x*+1/2, *y*−1/2, −*z*+1/2.
